# Exploring Focus and Depth-Induced Saliency Detection for Light Field

**DOI:** 10.3390/e25091336

**Published:** 2023-09-15

**Authors:** Yani Zhang, Fen Chen, Zongju Peng, Wenhui Zou, Changhe Zhang

**Affiliations:** 1 School of Electrical and Electronic Engineering, Chongqing University of Technology, Chongqing 400054, China; yani_zhang@stu.cqut.edu.cn (Y.Z.); pengzongju@126.com (Z.P.); 13614503112@163.com (C.Z.); 2 Faculty of Information Science and Engineering, Ningbo University, No. 818, Ningbo 315211, China; zouwench@163.com

**Keywords:** light field, saliency detection, focus cue, foreground, color and depth-induced cellular automata

## Abstract

An abundance of features in the light field has been demonstrated to be useful for saliency detection in complex scenes. However, bottom-up saliency detection models are limited in their ability to explore light field features. In this paper, we propose a light field saliency detection method that focuses on depth-induced saliency, which can more deeply explore the interactions between different cues. First, we localize a rough saliency region based on the compactness of color and depth. Then, the relationships among depth, focus, and salient objects are carefully investigated, and the focus cue of the focal stack is used to highlight the foreground objects. Meanwhile, the depth cue is utilized to refine the coarse salient objects. Furthermore, considering the consistency of color smoothing and depth space, an optimization model referred to as color and depth-induced cellular automata is improved to increase the accuracy of saliency maps. Finally, to avoid interference of redundant information, the mean absolute error is chosen as the indicator of the filter to obtain the best results. The experimental results on three public light field datasets show that the proposed method performs favorably against the state-of-the-art conventional light field saliency detection approaches and even light field saliency detection approaches based on deep learning.

## 1. Introduction

The light field is a densely sampled image array, which has brought humans closer to recording the real world. Compared with traditional images, the light field can record the intensity and direction of light rays and has a stronger expressive ability. With the development of refocusing and rendering techniques, many light field applications have been generated, such as depth estimation [1,2] and light field super-resolution [3,4]. As an important image preprocessing, light field saliency detection is crucial to promote the research of light field applications.

**Figure 1:**
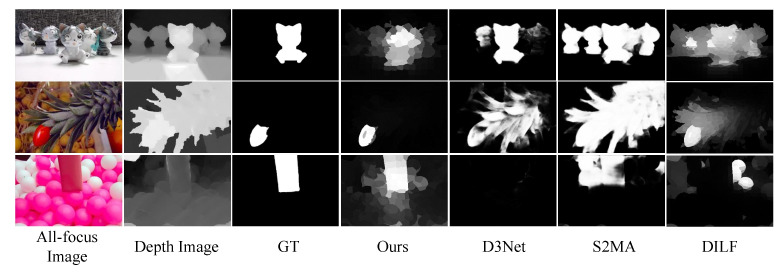
Comparison of the detection results due to depth image misleading by different methods (D3Net [21], S2MA [22], DILF [10]) on the DUT-LF [15] dataset.

Saliency detection aims to identify important regions that are interesting or obvious to human eyes and improve the understanding of computer vision applications, such as semantic segmentation [5], object detection [6], image recognition [7], etc. Saliency detection methods based on RGB images make it difficult to accurately detect salient objects in cluttered scenarios by color, compactness, or contrast cues. Moreover, saliency detection methods based on RGB-D images are easily misled by depth maps, as shown in Figure 1.

Existing methods [8,9,10,11,12,13,14] detected salient regions via hand-crafted features such as color, depth, and focus, while having a limited exploration of the light field and a few related studies. The deep learning methods, leveraging powerful extraction and expression capabilities, promote the development of light field saliency detection. Piao et al. [15] used a single sub-aperture image to synthesize the complex light field. In [16], a micro-lens image is used to predict salient regions through a convolutional network, which did not consider the correlation between sub-aperture maps. Wang et al. [17] designed a cross-modal feature fusion module to fuse the aggregated features from various modalities in the three networks of all-focus, depth, and focal stack images. Liang et al. [18] adopted a weakly supervised network and exploited the features of focal stack and depth maps to generate pixel-level pseudo-saliency maps. Jiang et al. [19] leveraged attention mechanisms to explore cross-modal complementarity and overcome information loss in the focal stack. Yuan et al. [20] refined the focal stack with depth modality, which enhances the structure and location information of salient objects in the focal stack.

However, there is no traditional method for exploring the relationship between the focal stack and the depth map, and how to use the interaction between these to improve the performance of saliency detection in the light field is still a problem worth thinking about.

The key to successfully identifying salient objects is via exploring the potential features and discovering the interactions between different cues in the light field. In this paper, we propose a light field saliency detection algorithm, in which the cues among the focal stack, depth, and all-focus images are fully explored and utilized to improve saliency detection via complementation and fusion. Specifically, the coarse region of the salient object is localized by combining the color and depth compactness. For the focal stack, we compute the background and foreground probabilities to highlight the foreground. Among these, the foreground object is enhanced by the depth contrast and the foreground probability. In addition, we introduce the local geodesic saliency cues [23] and combine them with the depth compactness to refine the saliency map. Furthermore, taking into account the consistency and smoothness of the saliency object, we design a saliency optimization model, the color and depth-induced cellular automata (CDCA), by exploiting the complementarity of the color and depth cues to optimize the salient detection map with higher accuracy. Finally, to avoid the influence of low-quality saliency maps, we use a filter to obtain excellent saliency detection results by comparing the values of the mean absolute error (MAE).

To summarize, the main contributions of this paper are:

1. We introduce a light field saliency detection method taking into account interactions among the depth, focus, color, and compactness cues.
2. We separate foreground and background by taking advantage of the focal stack and depth image. Exploring the depth feature, we extract the depth compactness and local saliency cues to emphasize local regions for refinement. At the same time, we integrate foreground probability with a depth contrast map to highlight the foreground.
3. We develop the CDCA optimization model, which integrates color and depth cues to improve the spatial consistency of saliency detection results.

The rest of the paper is organized as follows: Section 2 overviews the related works on RGB, RGB-D, and light field saliency detection. In Section 3, the proposed method and formulation are described in detail. The experimental results are presented and analyzed in Section 4. Finally, we summarize the proposed method in Section 5.

## 2. Related Works

This section will briefly review the related work on RGB images, RGB-D images, and light field saliency detection methods.

### 2.1. RGB Image Saliency Detection

RGB image saliency detection models can be divided into two categories, top-down and bottom-up models. Top-down methods are mainly task-oriented for saliency detection. Bottom-up methods mainly use the low-level features of an image, such as color, texture, and contrast information for saliency detection. This is especially true after the appearance of the simple linear iterative cluster (SLIC) [24] algorithm, which improves computational efficiency for image segmentation. Initially, saliency detection methods [25] used global and local contrast information as salient features for detection. Yang et al. [26] considered both foreground and background cues in a different way and ranked the similarity of the image regions via graph-based manifold ranking. Zhu et al. [27] analyzed the spatial distribution of the region and regarded the region with a large boundary border as the background. Zhou et al. [28] recovered the falsely suppressed salient regions by combining image internal compactness and local contrast information. Inspired by the automatic cellular machine, Yao et al. [29] used the propagation mechanism of the single-layer cellular automaton (SCA) to find the intrinsic correlation of similar regions and dynamically update the saliency map. At the same time, a multi-layer automatic cellular (MCA) optimization algorithm is proposed to integrate the advantages of the salient features.

It is difficult to achieve predictions that are highly consistent with human perception only by relying on low-level features, thus motivating many deep learning-based salient object detection models. Lou et al. [30] introduced a U-Net network to fuse multi-level context and perform salient object detection in both local and global manners. Wang et al. [31] added a pyramid attention structure and edge detection module to the network to obtain accurate salient area edges while expanding the receptive field. To make full use of the global context information, Chen et al. [32] utilized several context-aware modules to incorporate multiple levels of features with global contextual information. Since humans assign more attention to moving objects, Zhou et al. [33] proposed a motion-attention transfer network for zero-shot video object segmentation within the encoder, which not only inherits the advantages of multi-modal learning but also utilizes motion-attention to facilitate appearance learning. Fully supervised networks rely on a large amount of annotated data labels; Lai et al. [34] adopted weak supervision to improve salient objects in complex scenes by exploring the nature of visual attention patterns.

However, it is difficult to detect more accurate saliency results only by limiting features in challenging scenarios.

### 2.2. RGB-D Image Saliency Detection

According to the perceptual characteristics of human eyes, salient objects are often located in the foreground. Nowadays, many RGB-D saliency detection methods reduce misjudgments in challenging scenes by adding depth information. Niu et al. [35] demonstrated that stereoscopic information can provide a useful complement to existing RGB image saliency detection. Peng et al. [36] proposed a multi-stage RGB-D saliency detection model and demonstrated that depth information can improve the robustness of saliency results. Ren et al. [37] performed saliency detection by fusing global priors such as regional contrast, depth information, and background. Cong et al. [38] proposed a saliency detection method based on depth confidence and multi-information fusion to reduce the impact of depth images. Zhu et al. [39] found that the center-dark channel prior can distinguish the foreground and background. Cong et al. [23] refined the generated saliency detection map by extracting the shape and contour of salient objects in the depth image and achieved excellent results.

In addition, the method of RGB-D saliency detection using deep learning [21,22,40,41] can achieve better results. Zhu et al. [40] designed an independent sub-network to extract depth cues and guide the main network to improve the saliency detection performance. Liu et al. [22] proposed a selective attention mechanism to weigh the mutual attention to filter unreliable information. Fan et al. [21] proposed a new RGB-D dataset and designed a deep cleaning unit to filter low-quality salient results. Zhao et al. [41] designed a depth-awareness framework by excavating depth information and exploiting the low-level boundary cues to achieve accurate salient detection results. However, RGB-D image unavoidably suffer from the influence of poor-quality depth images on the detection results, especially when the salient and non-salient objects are at the same depth.

### 2.3. Light Field Saliency Detection

The rich features of the light field are beginning to be used to supplement the insufficiency of RGB and RGB-D images. Li et al. [8] calculated the likelihood score for the first time to distinguish the foreground and background of light field images for saliency detection. Li et al. [9] constructed salient and non-salient dictionaries from feature vectors, but the salient results could not provide a better visual experience for human eyes. Zhang et al. [10] combined depth, color contrast, and background probability for saliency detection and obtained the complete salient region. Wang et al. [11] fused color contrast, background prior, depth, and focus information through a Bayesian framework, but did not fully explore the depth image. Zhang et al. [12] used multiple cues to improve the performance of saliency detection by complementing the differences between different information. The single-layer cellular automaton can enhance the saliency consistency between similar regions. Inspired by [29], Piao et al. [13] proposed a depth-guided automatic cellular machine model (DCA) that can automatically optimize saliency maps based on depth, focus, and color information. Wang et al. [14] calculated the degree of focus to generate depth information to reduce the dependence on the depth map.

With the continuous improvement of light field datasets, many models have begun to use convolutional networks to extract the salient features of the light field. Piao et al. [15] proposed a multi-view object detection network to synthesize multi-view images for saliency detection. Zhang et al. [16] proposed an end-to-end convolutional network to extract the salient features of micro-lens images. Wang et al. [42] and Zhang et al. [43] have committed to exploring the correlation and fusion between focal stack and all-focus images to improve saliency detection performance, but they need to rely on high-quality focal stacks. To aggregate cross-level features, Wang et al. [17] propose a cross-modal feature fusion module to fuse features from various modalities from three sub-networks. Liang et al. [18] designed a weakly supervised learning framework based on a pseudo-ground truth to solve the problem of unclear edges of salient objects in complex structures. Jiang et al. [19] utilized the attention mechanism to explore cross-modal complementarities and generated object edges and edge features to progressively refine regional features to achieve edge-aware detection. Zhang et al. [44] designed a multi-task collaborative network for light field salient object detection to explore the complementary coherence among multiple cues, including spatial, edge, and depth information. Feng et al. [45] exploited the relationship between light field cues to identify clean labels from pixel-level noisy labels for saliency detection. Yuan et al. [20] used the multi-modal feature guidance method to refine the focal stack, enhance the structure and position information about the salient objects in the focal stack, and improve accuracy.

Compared with conventional methods, deep learning methods can obtain high-quality and remarkable results but require greater computing power, which increases the cost of experiments to a certain extent. We focus extensively on designing a low-cost detection model and adopting the interaction and complementarity between light field cues to improve the saliency detection performance in challenging scenarios.

## 3. Methodology

**Figure 2:**
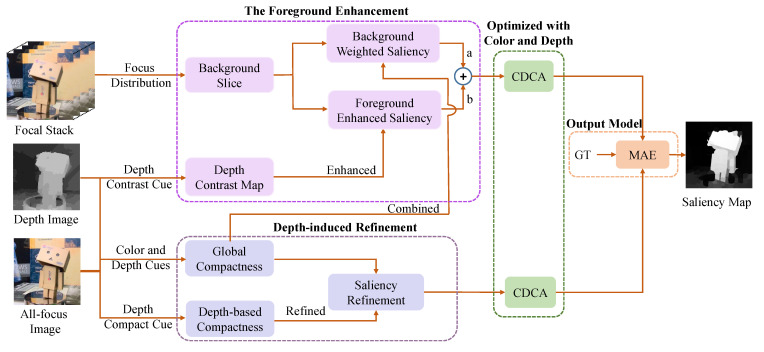
The framework of the proposed method where “a” and “b” are the different weighted coefficient to balance the background saliency map and foreground saliency map.

**Figure 3:**
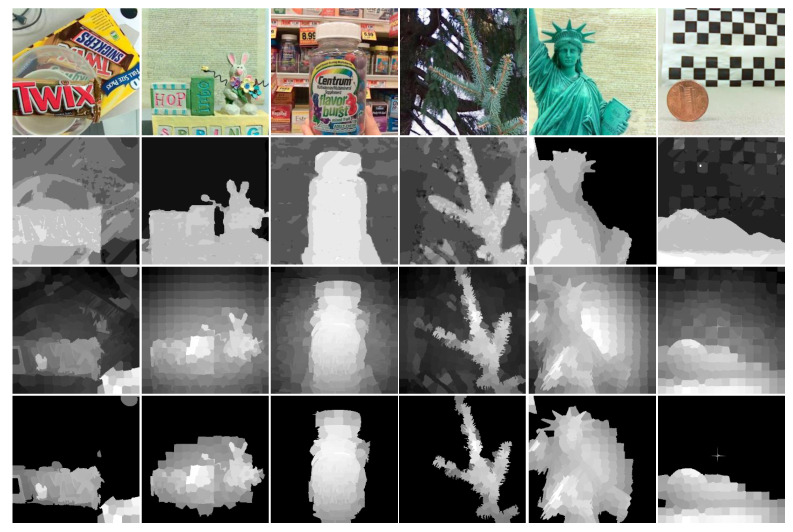
Foreground probability enhances depth contrast saliency map (from top to bottom are the all-focus image, depth image, depth contrast saliency map, and enhancement result).

**Figure 4:**
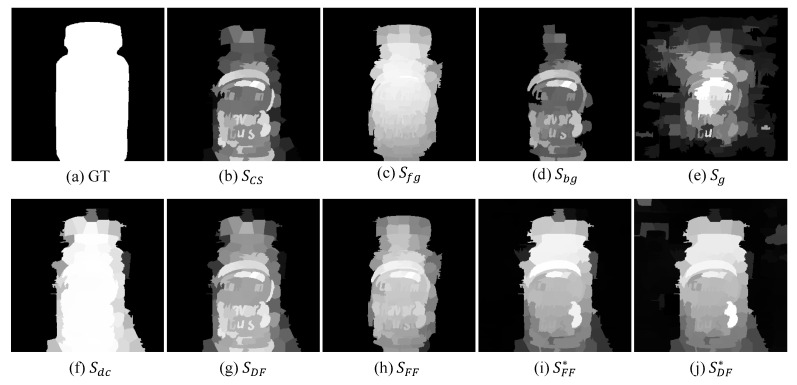
The visual process of the proposed method.

In this paper, we make full use of the advantages of focus, depth, and color to improve the accuracy of the saliency detection model. Figure 2 shows the framework of the proposed method and the framework mainly has the following stages: (1) We obtain the global compactness saliency to locate the compactly distributed areas. (2) The foreground and background probabilities are calculated and respectively combined with the depth contrast cue and the global compactness map to highlight the salient objects, as shown in Figure 3. (3) The global compactness saliency is refined by exploring high-quality depth compactness and local geodesic cues. (4) The saliency maps are optimized by the CDCA model to obtain a more perfect saliency map. (5) We design an output model to obtain excellent saliency detection results by judging the MAE value. Figure 4 shows the visual processes of each step. In the next section, this paper presents the details of the method.

### 3.1. Compactness Based on Color and Depth Cues

In the spatial domain, the salient region usually has a compact spread. In the depth domain, the region closest to the camera often contains a concentrated distribution in the depth image. Motivated by this, we integrate color and depth to define global compactness, which can be used to distinguish salient objects from the background.

We divide an image into compact and homogenous superpixels by the SLIC algorithm [24] and construct a graph G=(V,E), where *V* represents the generated superpixel node set, and *E* represents the distance between adjacent nodes’ connection set. Therefore, the similarity between superpixels $v_{i}$ and $v_{j}$ in the Lab color space and depth space is defined as:

(1) $$a_{ij}^{c}=exp(-||c_{i}-c_{j}{\left|\right|}_{2}/\sigma^{2})$$

(2) $$a_{ij}^{d}=exp(-\lambda_{d}\cdot |d_{i}-d_{j}\left|/\sigma^{2}\right)$$

where $c_{i}$ is the mean color value of $v_{i}$ superpixels in the Lab color space, the mean depth value of $v_{i}$ superpixels in $d_{i}$ depth space, $\lambda_{d}=exp\left(\left(1-m_{d}\right)\cdot CV\cdot H-1\right)$ is the depth confidence [38], $m_{d}$ is the mean value of the depth map, CV is the coefficient of variation, and H denotes the depth frequency entropy. $\lambda_{d}$ is used to judge the quality of a depth image. The higher the value of $\lambda_{d}$, the better the quality of the depth image. $\sigma^{2}=0.1$ [26] is a constant that controls the strength of the similarity. Here, the global compactness based on color and depth is defined as:

(3) $$S_{CS}\left(i\right)=1-norm\left(cc\left(i\right)+cd\left(i\right)\right)$$

where $norm\left(x\right)=\left(x-x_{min})/(x_{max}-x_{min}\right)$ is a function that normalizes x to the range of [0, 1]. cc(i) and cd(i) are the color compactness and depth compactness of the superpixel $v_{i}$, respectively. They are expressed as follows:

(4) $$cc\left(i\right)=\frac{\sum_{j=1}^{N}n_{j}\cdot a_{ij}^{c}\cdot ∥b_{j}-\mu_{i}∥}{\sum_{j=1}^{N}n_{j}\cdot \left(a_{ij}^{c}+a_{ij}^{d}\right)}$$

(5) $$cd\left(i\right)=\frac{\sum_{j=1}^{N}n_{j}\cdot a_{ij}^{d}\cdot ∥b_{j}-p_{0}∥}{\sum_{j=1}^{N}n_{j}\cdot \left(a_{ij}^{c}+a_{ij}^{d}\right)}$$

where *N* represents the number of superpixels, $n_{j}$ represents the number of pixels in the superpixel $v_{i}$, $b_{j}=\left[b_{j}^{x},b_{j}^{y}\right]$ represents the centroid coordinates of the superpixel $v_{j}$, $\mu_{i}=\left[\frac{\sum_{j=1}^{N}a_{ij}{}^{c}\cdot n_{j}\cdot b_{j}^{x}}{\sum_{j=1}^{N}a_{ij}{}^{c}\cdot n_{j}},\frac{\sum_{j=1}^{N}a_{ij}{}^{c}\cdot n_{j}\cdot b_{j}^{y}}{\sum_{j=1}^{N}a_{ij}{}^{c}\cdot n_{j}}\right]$ represents the spatial average value, and $p_{0}$ is the coordinate of the center.

### 3.2. Exploring Focus for Foreground Enhancement

Effectively distinguishing the foreground from the background is a key step in salient detection. Considering the problem that salient objects are mostly located in the foreground and the foreground is not easy to obtain, we analyze the focus distribution in the focal stack to select the background slice, and determine the foreground by finding the background. At the same time, global compact saliency maps based on color and depth information can comprehensively detect salient objects in images, but the detection results are rough. To further suppress the interference caused by the background, we fuse the background and foreground probabilities with the global compact map and the depth-contrast saliency map, respectively, to achieve the purpose of separating the foreground and background.

The focal stack is a set of focused slices focused on the foreground and background, and the difference of the focus point will lead to the sharpness difference of different regions. Considering the advantages of the center prior and background prior, we detect background regions and compute background and foreground probabilities to highlight salient objects.

In order to highlight the foreground and suppress the background, we select the background slice and compute the background probability [10] to refine the global compactness saliency map, and the result $S_{bg}\left(i\right)$ is as follows:

(6) $$S_{bg}\left(i\right)=\sum_{i=1}^{N}S_{cs}\left(i\right)\cdot Pb_{bg}\left(i\right)$$

(7) $$Pb_{bg}\left(i\right)=1-exp\left(-\frac{U_{bg}{\left(i\right)}^{2}}{2\sigma_{bg}^{2}}\cdot ∥p_{0}-U_{pos}^{*}\left(i\right)∥{}^{2}\right)$$

where we set $\sigma_{B}$ = 1 to ensure that the background probability is maximized, $U_{foc}\left(i\right)$ is the mean value of superpixel $v_{i}$ of the slice, $||C-U_{pos}^{*}\left(i\right)\left|\right|$ is used to measure the superpixel spatial information related to the superpixel and the image center, and $U_{pos}^{*}\left(i\right)$ is the normalized average coordinate of the superpixel $v_{i}$.

At the same time, to fully extract the depth cues, we introduce the foreground probability $P_{fg}\left(i\right)$ [13] to highlight the foreground objects. Figure 3 shows that the foreground probability enhances the depth cues. The foreground saliency map $S_{fg}\left(i\right)$, induced by the depth cues, is defined as:

(8) $$S_{fg}\left(i\right)=\sum_{j=1}^{N}S_{D}\left(i\right)\cdot P_{fg}\left(j\right)$$

(9) $$P_{fg}\left(i\right)=exp\left(-\frac{U_{foc}{\left(i\right)}^{2}}{2\sigma_{F}^{2}}\cdot {∥p_{0}-U_{pos}^{*}\left(i\right)∥}^{2}\cdot {∥1-d(i)∥}^{2}\right)$$

where $\sigma_{F}$= 0.2, and the depth-induced contrast saliency $S_{D}$ and spatial weight factors are:

(10) $$S_{D}\left(i\right)=\sum_{j=1}^{N}W_{pos}\left(i,j\right)\cdot ∥d_{i}-d_{j}∥$$

(11) $$W_{pos}\left(i,j\right)=exp\left(-\frac{\left|\right|U_{pos}^{*}\left(i\right)-U_{pos}^{*}\left(j\right){\left|\right|}^{2}}{2\sigma_{w}^{2}}\right)$$

where $\sigma_{w}$= 0.67.

A depth prior has a great help in distinguishing a salient object from the background. To further emphasize the salient object, we distribute more weight to the foreground salient maps, and the saliency detection result map $S_{FF}$ is obtained by weighted fusion as:

(12) $$S_{FF}=\alpha \cdot S_{fg}+\left(1-\alpha \right)\cdot S_{bg}$$

where α is set to 0.7.

### 3.3. Depth-Induced Refinement

In order to avoid the influence of a poor-quality depth image, the depth confidence [38] is introduced to calculate the high-quality depth compactness and refine the foreground. The improved depth compactness $S_{dc}\left(i\right)$ is defined as:

(13) $$S_{dc}\left(i\right)=1-N\left(\frac{\sum_{j=1}^{N}n_{j}\cdot a_{ij}^{c}\cdot a_{ij}^{d}\cdot ∥b_{j}-p_{0}∥\cdot exp\left(-\frac{\lambda_{d}\cdot d_{i}}{\sigma^{2}}\right)}{\sum_{j=1}^{N}n_{j}\cdot a_{ij}^{c}\cdot a_{ij}^{d}}\right)$$

Observing the depth map contained in existing light field datasets, we find that the quality of the depth map correlates with the detection of high-quality salient objects. Considering the quality of depth maps of different datasets, we adopt the average depth confidence value as a benchmark to extract depth information. When the quality of the depth map is reliable (i.e., $\lambda_{d}\geq mean$), the salient object has an obvious depth contrast with the background, and the depth compactness can refine the saliency map. When the quality of the depth map is poor (i.e., $\lambda_{d}<mean$), we utilize the global compactness to strengthen the salient features. For refinement, the saliency map combined with the depth feature is defined as:

(14) $$S_{DF}\left(i\right)=\left\{\begin{array}{cc} \; & 0.5\cdot S_{CS}\left(i\right)+0.5\cdot N\left(S_{dc}\left(i\right)\cdot S_{g}\left(i\right)+S_{dc}\left(i\right)\right),\lambda_{d}\geq mean\\ \; & 0.5\cdot S_{CS}\left(i\right)+0.5\cdot N\left(S_{dc}\left(i\right)\cdot S_{CS}\left(i\right)+S_{dc}\left(i\right)\right),\lambda_{d}<mean \end{array}\right.$$

where the local geodesic distance saliency $S_{g}\left(i\right)$ [23] is introduced to accumulate edge weights along the shortest path from a superpixel to a background node, mean is the average depth confidence of the depth image, and the specific value is placed in the experiment section.

### 3.4. Color and Depth-Induced Optimization

We observe that the edges of the generated saliency maps are not clear, the salient objects are incomplete, and the background still has subtle disturbances. Considering the consistency of color smoothing and depth space, the proposed algorithm adds depth information on the basis of the existing optimization model, and the improved optimization model can obtain salient detection results with clearer edges and improved quality.

To obtain a meticulous saliency map, we improve upon a model called the color and depth-induced cellular automata (CDCA) to optimize saliency maps. The CDCA model is mainly based on two considerations: (1) Depth information helps highlight foreground objects. We integrate the color and depth cues to define cell neighbors can improve the optimization accuracy. (2) To avoid the influence of poor-quality depth maps, superpixels on the image boundaries are considered background seeds. When the quality of the depth map is reliable, the CDCA model can effectively optimize the saliency map, and its synchronous update rule can greatly improve the detection of incomplete prior saliency maps in challenging scenes. Therefore, we construct the impact factor matrix $f_{ij}^{cd}$ as follows:

(15) $$f_{ij}^{cd}=\left\{\begin{array}{cc} \; & exp(-(||c_{i}-c_{j}{\left|\right|}_{2}+|d_{i}-d_{j}\left|\right)/\sigma^{2}),j\epsilon NB\left(i\right)\\ \; & 0,i=j\;or\;otherwise \end{array}\right.$$

where $\sigma^{2}$ is a parameter controlling the strength of similarity, and set $\sigma^{2}$= 0.1. NB(i) is the set of the neighbors of cell *i*. To normalize the influence factor matrix, we generate the degree matrix $D=diag\left\{d_{1},d_{2},\cdots ,d_{N2}\right\}$, $d_{i}=\sum_{j}^{}f_{ij}^{cd}$. Then, the normalized influence factor matrix is:

(16) $$F^{*}=D^{-1}\cdot F$$

In order to balance the importance of the cell’s current state and the cell’s neighbors’ state, a coherence matrix $C=diag\left\{c_{1},c_{2},\cdots ,c_{N}\right\}$ is constructed to promote the evolution of the cell. Then, the consistency calculation of each cell to its current state is:

(17) $$c_{i}=\frac{1}{max(f_{ij})}$$

In order to control $c_{i}$ in the range of [b,a+b], the constants *a* and *b* are set to 0.6 and 0.2, respectively. Then, the coherence matrix is $C{}^{*}=diag\left\{c_{1}^{*},c_{2}^{*},\cdots ,c_{N}^{*}\right\}$ as:

(18) $$c_{i}^{*}=a\cdot \frac{c_{i}-min\left(c_{j}\right)}{max\left(c_{i}\right)-max\left(c_{j}\right)}+b$$

Here, the synchronous update rule is defined as:

(19) $$S^{t+1}=C^{*}\cdot S^{t}+\left(I-C^{*}\right)\cdot F^{*}\cdot S^{*}$$

where $S^{t}$ is the refined saliency map when *t*= 0, and the ultimate saliency map after N1 time steps is denoted as $S^{t+1}$.

### 3.5. Output Model Using MAE

To reduce the redundant information brought by saliency map fusion, the output result mainly considers the following aspects: (1) When the quality of the depth image is poor, the noise introduced by the low-quality depth map should be avoided. (2) When the quality of the depth image is reliable, salient objects can be identified by depth contrast cues. (3) When the focal stack and the depth image are both reliable, we filter to obtain the excellent saliency results. Figure 4 shows the visual process of the proposed method.

We design a simple screening filter, judging the mean absolute error (MAE) value, to obtain the optimal prediction result. The higher the saliency map accuracy is, the smaller the value of the MAE is. The final saliency detection result is denoted as:

(20) $$S_{LF}=\left\{\begin{array}{cc} \; & S_{DF},MAE_{S_{DF}}\leq MAE_{S_{FF}}\\ \; & S_{FF},MAE_{S_{DF}}>MAE_{S_{FF}} \end{array}\right.$$

## 4. Experiment and Verification

### 4.1. Dataset and Parameter Setup

In this paper, we select the existing public light field datasets, LFSD [8], HFUT [12], and DUT-LF [15] to verify the effectiveness and robustness of the proposed method. The LFSD dataset captures 60 indoor and 40 outdoor scenes, most of which have a single salient object and reliable depth image quality. The HFUT dataset contains 255 pictures, which not only contains a large number of challenging scenes, such as small objects, multiple targets, or image blur, but also the depth images are poor quality. The DUTLF-FS dataset consists of 1000 training images and 462 test images. The salient object has the characteristics of small size, low contrast with the background, and multiple salient objects without connection. At the same time, some images are affected by light intensity. In the experiments, we use test images of the DUT-LF dataset for verification and comparison, and the experiments run on Matlab 2018b.

We set the number of superpixels to 200 in all experiments. In the CDCA model, the number of time steps N1 = 20. In the depth refinement stage, the average depth confidence of LFSD [8] and DUT-LF [15] is 0.22, and that of HFUT [12] is 0.03.

### 4.2. Performance Evaluation Measures

To conduct a quantitative performance evaluation, we compute the precision-recall (PR) curve, F-measure, WF-measure [46], E-measure [47], S-measure [48], and mean absolute error (MAE) to evaluate the state-of-the-art detection models used for comparison.

The PR curve reflects the relationship between precision and recall. By binarizing the saliency map and the ground-truth map with a threshold, the value of precision and recall can be calculated. The F-measure is the result calculated by the weighted sum of the precision and recall. The higher the F-measure value is, the more effective the model is:

(21) $$F_{\beta }=\frac{(1+\beta^{2})\cdot Precision\cdot Recall}{\beta^{2}\cdot Precision+Recall}$$

where $\beta^{2}$ = 0.3.

In [46], considering the correlation between the pixels of the saliency map and the position of the wrong pixels, a weighting function ω is added in precision and recall to represent the importance of the pixels and the degree of dependence between different pixels. The weighted F-measure is defined as follows:

(22) $$F_{\beta }^{\omega }=\frac{\left(\left(1+\beta^{2}\right)Precision^{\omega }\cdot Recall^{\omega }\right)}{\left(\beta^{2}\cdot Precision^{\omega }+Recall^{\omega }\right)}$$

The E-measure is used to evaluate the structural similarity between the saliency detection map and the ground-truth map [47], and its specific formula is:

(23) $$E_{S}=\frac{1}{W\cdot H}\sum_{x=1}^{W}\sum_{y=1}^{H}\phi \left(x,y\right)$$

where ϕ(x,y) is the enhanced alignment matrix.

The S-measure is used to obtain the two characteristics of pixel-level matching and image-level statistics [48], and its specific formula is:

(24) $$S_{\lambda }=\lambda \cdot S_{o}+\left(1-\lambda \right)\cdot S_{\gamma }$$

where $S_{o}$ and $S_{\gamma }$ represent object-aware and region-aware structural similarity, respectively, and λ is a balance parameter and is set to 0.5.

The MAE expresses the similarity between the saliency map and the true value map, which is used to measure the average error between each pixel of the binarized saliency map and the ground truth. The MAE is expressed as follows:

(25) $$MAE=\frac{1}{W\cdot H}\sum_{x=1}^{W}\sum_{y=1}^{H}∥S(x,y)-GT(x,y)∥$$

where *W* and *H*, respectively, represent the width and height of the image, S(x,y) is the continuous saliency map, and GT(x,y) is the the binary ground truth.

### 4.3. Comparison with State-of-the-Art Methods

**Figure 5:**
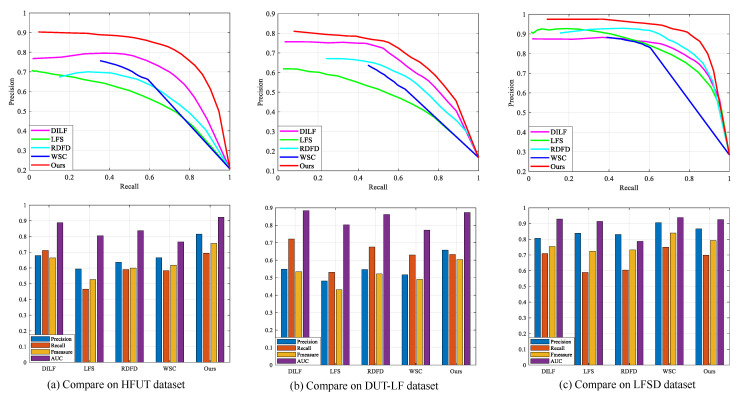
Performance comparison with the proposed method and the state-of-the-art conventional light field saliency detection methods.

In this paper, we focus on bottom-up saliency detection models and qualitatively compare them with the state-of-the-art conventional light field methods (LFS [8], WSC [9], DILF [10], and RDFD [14]). All comparative saliency maps were provided by the authors or run on publicly available code. Figure 5 shows the visual comparison of the proposed method with the others on the LFSD [8], HFUT [12], and DUT-LF [15] datasets, and the proposed method achieves the highest PR curve. On the HFUT and DUT-LF datasets shown in Figure 5a,b, the proposed method can improve the detection performance in challenging scenes by exploring the interaction and complementarity among different light field features when the quality of the depth map and focal stack is poor. When the quality of the image is reliable, the proposed method can achieve the superior saliency detection performance shown in Figure 5c.

Table 1 shows the quantitative performance evaluation of the proposed method and the others (LFS [8], WSC [9], DILF [10], RDFD [14]). The proposed method achieves the best score, and the saliency detection results obtained are superior to the latest conventional methods. It is demonstrated that salient object detection performance in challenging scenarios can be improved by exploiting the interaction and complementarity among the salient features of the light field.

**Figure 6:**
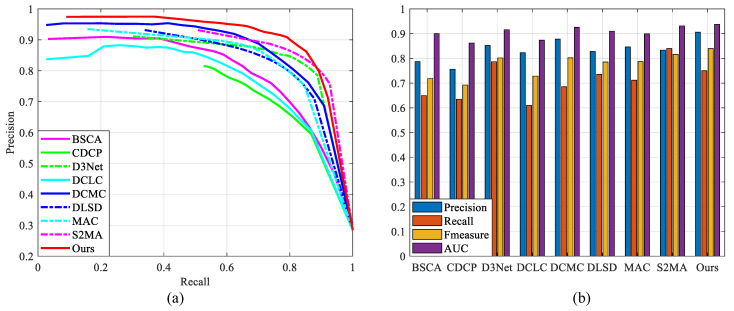
Performance comparison of the proposed method with other state-of-the-art saliency detection methods (RGB/RGB-D/light field) on LFSD dataset (the dotted line is the deep learning method). (**a**) Precision–recall curves of different methods. (**b**) Average precision, recall, F -measure, and AUC of different methods.

To prove that the proposed method is better than other state-of-the-art saliency detection methods, we compare them with related RGB/RGB-D and light field methods, including RGB conventional methods (DCLC [28], BSCA [29]), RGB-D methods (CDCP [39], DCMC [38], D3Net [21], S2MA [22], PDNet [40]), and light field methods (DLSD [15], MAC [16], DCA [13], NoiseLF [45]). The experiments show that the proposed method not only outperforms conventional saliency detection methods but also achieves a more accurate salient object detection at a lower computational cost than deep learning methods, as shown in Table 2 and Figure 6.

### 4.4. Ablation Study

In this section, we analyze the effectiveness of using depth and color cues in the CDCA model and the contributions of different components of the proposed method.

#### 4.4.1. The Effectiveness of the CDCA Model

To prove the effectiveness of the CDCA model in the proposed method, we compare the results of utilizing the CDCA model and non-optimization, respectively. The experimental results show that the saliency map generated by the CDCA model is closer to the ground truth with a clearer edge. Table 3 demonstrates that the CDCA model helps improve the performance of saliency detection. The weighted F-measure considers the correlation between the pixels of the saliency map, while the saliency map that has not been optimized by the CDCA model contains more redundant information, which increases the correlation between pixels.

**Figure 7:**
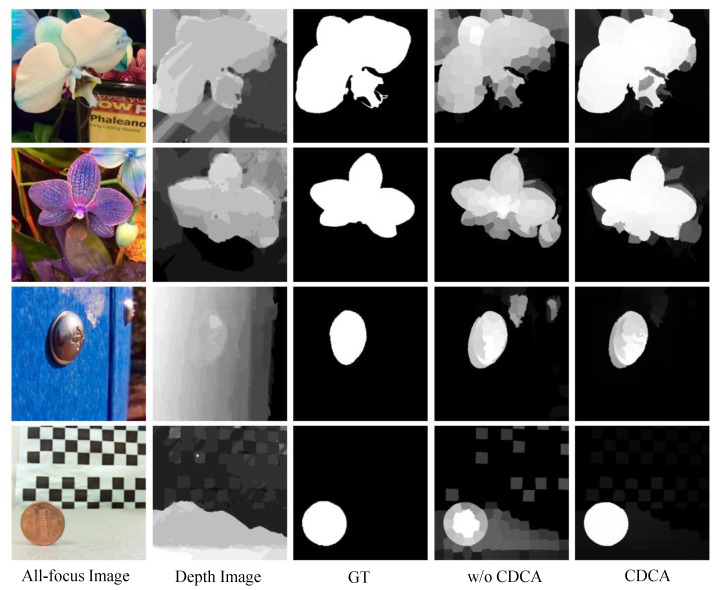
The comparison of the saliency maps after optimization by the proposed CDCA model and without the CDCA model on the LFSD dataset.

Figure 7 shows that the depth feature can correct the saliency map when there are complex colors in the scenes (rows 1 and 2). When the depth image cannot highlight the salient objects (rows 3 and 4), the color contrast cue can be complemented with the depth, resulting in a clearer salient result. Through quantitative and qualitative analyses, the CDCA model can improve the accuracy of saliency detection by utilizing the complementarity of color and depth.

To verify the superiority of the CDCA model and explain the difference between the proposed CDCA model and the SCA [29] model, we compare the corresponding saliency map (generated by LFS [8], DILF [10], WSC [9], and RDFD [14]) optimized by the CDCA model and the SCA model on the LFSD [8] dataset. The suffix with CDCA in Table 4 denotes the saliency map optimized by the CDCA model. As shown in Table 4, the LFS method has the most obvious effect after optimization; the F-measure increased by 10.6%, and the MAE decreased by 29.68%. The CDCA model, which adds color and depth cues to increase spatial consistency, optimizes the saliency maps with a higher precision. Therefore, the CDCA model is suitable for light field images and has a strong generalization.

**Figure 8:**
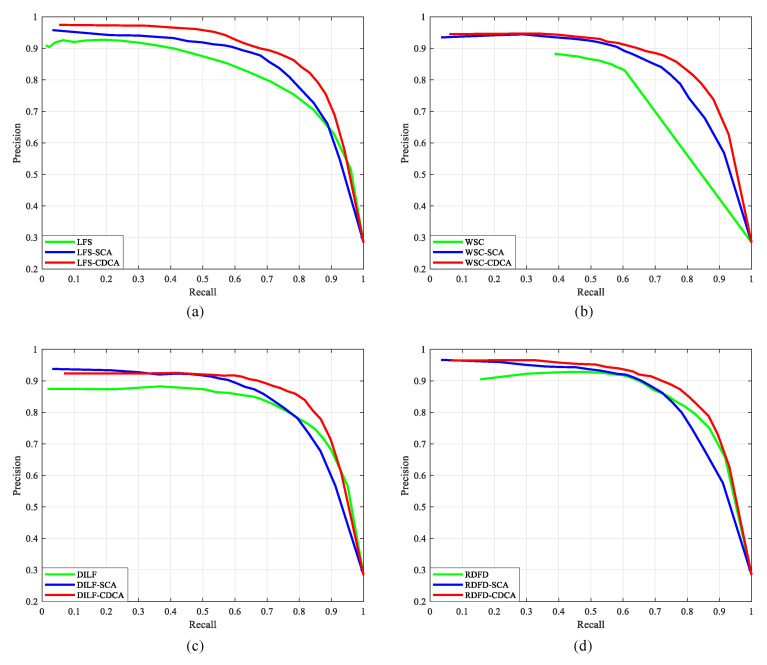
The comparison of the PR curves after optimization by the proposed CDCA model and the SCA model on the LFSD dataset. (**a**) LFS method. (**b**) WSC method. (**c**) DILF method. (**d**) RDFD method.

**Figure 9:**
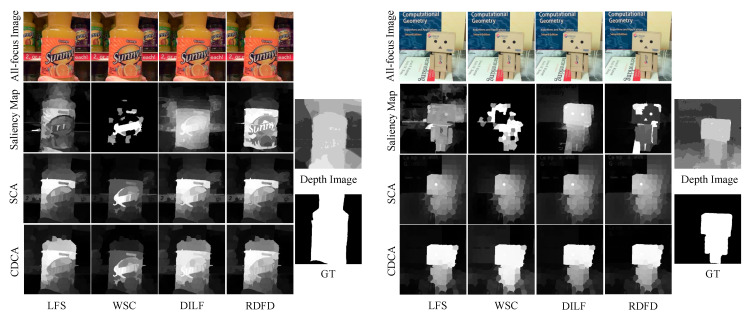
The visual comparison of the saliency maps after optimization by the proposed CDCA model and the SCA model on the LFSD dataset.

Figure 8 shows the performance comparison of the existing light field saliency detection methods after optimization of the SCA [29] and CDCA models on the LFSD [8] dataset. The PR curve shows that the CDCA model achieves a higher PR curve compared to the SCA model. Figure 9 shows the visual comparison of the optimized saliency map between the CDCA and SCA models on the LFSD [8] dataset. It is observed that when the background color is similar to the salient object, the CDCA model can optimize the more accurate salient detection results because of the depth feature. In the case of a reliable depth image, the CDCA model can obtain more accurate salient edges. It is undeniable that the CDCA model is susceptible to the impact of poor-quality depth images. However, combining color and depth information compensates for the negative impact of the depth image quality to a certain extent.

#### 4.4.2. The Effectiveness of the Focal Stack and Depth

To verify the effectiveness of interaction and complementarity between different light field cues, we evaluate a variant of the proposed method by sequentially joining the focal stack and the depth map on the LFSD [8] dataset, as shown in Table 5. The focal stack and depth image have improved all indicators, and it is also proved that the CDCA model can obtain higher-precision saliency detection results.

**Figure 10:**
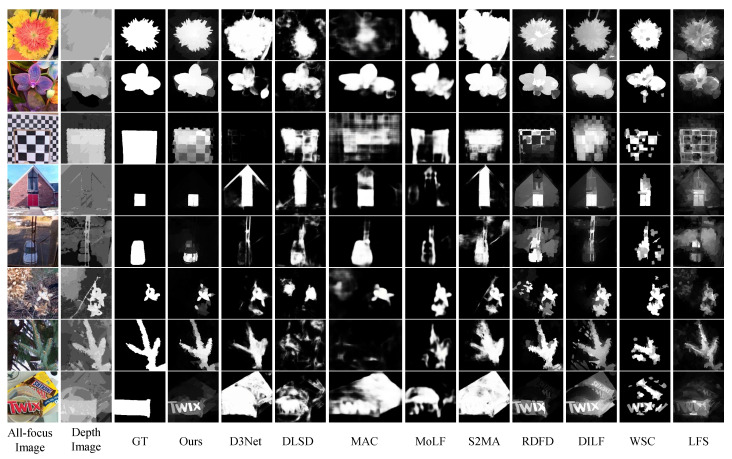
The visual comparison of our saliency maps with other state-of-the-art methods on LFSD dataset.

Figure 10 shows the visual comparison between the proposed method and others on the LFSD [8] dataset. The foreground and background in the all-focus images are relatively similar (rows 2, 3, 6, 8), and the salient objects can be effectively identified with the supplement of depth information. The depth images in the fourth and fifth rows can easily mislead detection, while focus and color cues can play a corrective role.

**Figure 11:**
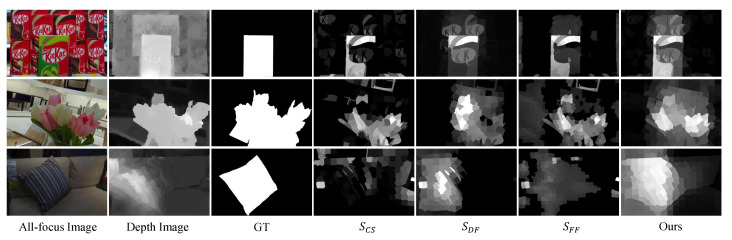
Some failure results by the proposed method.

As illustrated in Figure 11, we also exhibit some failures brought by the proposed method. The performance of the method is partially dependent on the accuracy of the depth map and the focus region of the focal stack. If the depth map is seriously blurred or amorphous, it yields incorrect results. If the foreground and background are similar in color and disorderly, it is necessary to rely on depth compactness and focus to extract and detect salient objects. Therefore, obtaining an accurate depth image as well as a perfect focal region of the focal stack remains a challenging problem.

## 5. Conclusions

In this paper, we propose a light field saliency detection method based on focus and depth, which explores the interaction and complementarity among focus, color, and depth cues of the light field to improve the saliency detection performance. Firstly, coarse salient regions are localized by combining the compactness of color and depth. Then, the interplay of depth and focus information is used to highlight the foreground and suppress the background. At the same time, the local depth cue is used to enhance the global features to refine the salient map. Secondly, inspired by spatial consistency, we utilize the complementarity of color and depth information to improve previous optimization models, resulting in remarkable results with higher accuracy. Finally, to avoid the influence of image quality, we design an output model with the MAE as the screening index.

The proposed method can obtain high-quality saliency detection results with lower computational cost by deeply exploring different cues of the light field. According to the comprehensive comparison of public datasets and ablation experiments, it is proved that the proposed method is far superior to the conventional light field saliency detection methods and even better than some state-of-the-art methods based on deep learning.

## Figures and Tables

**Table 1:** Quantitative comparison between the proposed method and the state-of-the-art saliency detection methods on different datasets (E-measure ($E_{\beta }$), S-measure ($S_{\alpha }$), WF-measure ($F_{\beta }^{\omega }$), F-measure ($F_{\beta }$), and MAE(*M*)) (bold: best).

Method	LFSD [8]					HFUT [12]					DUT-LF [15]				
	$E_{\beta }$	$S_{\alpha }$	$F_{\beta }^{\omega }$	$F_{\beta }$	M	$E_{\beta }$	$S_{\alpha }$	$F_{\beta }^{\omega }$	$F_{\beta }$	M	$E_{\beta }$	$S_{\alpha }$	$F_{\beta }^{\omega }$	$F_{\beta }$	M
LFS [8]	0.749	0.660	0.470	0.725	0.219	0.666	0.565	0.260	0.426	0.222	0.742	0.585	0.309	0.525	0.228
WSC [9]	0.778	0.693	0.637	0.735	0.163	0.679	0.613	0.428	0.485	0.154	0.787	0.656	0.527	0.617	0.151
DILF [10]	0.828	0.790	0.654	0.787	0.149	0.693	0.672	0.430	0.530	0.151	0.813	0.725	0.517	0.663	0.157
RDFD [14]	0.813	0.760	0.647	0.792	0.152	0.691	0.619	0.355	0.518	0.215	0.782	0.658	0.443	0.599	0.192
Ours	**0.847**	**0.812**	**0.720**	**0.840**	**0.124**	**0.746**	**0.687**	**0.455**	**0.600**	**0.148**	**0.841**	**0.759**	**0.557**	**0.756**	**0.144**

**Table 2:** Quantitative comparisons between the proposed method and the state-of-the-art deep learning and traditional methods on the LFSD dataset ((E-measure ($E_{\beta }$), S-measure ($S_{\alpha }$), WF-measure ($F_{\beta }^{\omega }$), F-measure ($F_{\beta }$), and MAE(*M*)). The top three models are highlighted in red, blue, and green, respectively.

Category	Method	$E_{\beta }$	$S_{\alpha }$	$F_{\beta }^{\omega }$	$F_{\beta }$	*M*
RGB	DCLC [28]	0.765	0.668	0.511	0.728	0.200
	BSCA [29]	0.780	0.723	0.549	0.719	0.198
RGB-D	CDCP [39]	0.763	0.699	0.595	0.692	0.181
	DCMC [38]	0.817	0.722	0.584	0.802	0.172
RGB-D${}^{DL}$	D3Net [21]	0.840	0.808	0.751	0.802	0.107
	S2MA [22]	0.851	0.820	0.764	0.816	0.105
	PDNet [40]	-	-	-	0.822	0.075
Light Field${}^{DL}$	DLSD [15]	0.840	0.778	0.703	0.785	0.125
	MAC [16]	0.819	0.768	0.681	0.787	0.133
	NoiseLF [45]	-	-	-	0.804	0.111
Light Field	DCA [13]	-	-	-	0.831	0.133
	Ours	0.847	0.812	0.720	0.840	0.124

**Table 3:** The effectiveness of the CDCA model in the proposed method on the LFSD dataset ($E_{\beta }$), S-measure ($S_{\alpha }$), WF-measure ($F_{\beta }^{\omega }$), F-measure ($F_{\beta }$), and MAE(*M*)) (bold: best).

Method	$E_{\beta }$	$S_{\alpha }$	$F_{\beta }^{\omega }$	$F_{\beta }$	*M*
Compactness	0.801	0.700	0.577	0.743	0.178
w/o CDCA	0.844	0.797	**0.721**	0.831	0.125
+ CDCA	**0.847**	**0.812**	0.720	**0.840**	**0.124**

**Table 4:** Performance comparison between the proposed method and the light field saliency detection methods after the CDCA optimization (F-measure ($F_{\beta }$) (bold: best).

Method	Ours	LFS	LFS-CDCA	DILF	DILF-CDCA	WSC	WSC-CDCA	RDFD	RDFD-CDCA
$F_{\beta }$	**0.840**	0.725	0.802	0.787	0.814	0.735	0.800	0.792	0.820
MAE	**0.124**	0.219	0.154	0.149	0.149	0.163	0.154	0.152	0.147

**Table 5:** Performance comparison of each component in the whole algorithm where FocalStack+ represents the contribution of salient features in the focal stack and Depth+ represents the contribution of depth cue to the model. ((E-measure ($E_{\beta }$), S-measure ($S_{\alpha }$), WF-measure ($F_{\beta }^{\omega }$), F-measure ($F_{\beta }$), and MAE(*M*)) (bold: best).

Settings	$E_{\beta }$	$S_{\alpha }$	$F_{\beta }^{\omega }$	$F_{\beta }$	*M*
Compactness	0.801	0.700	0.577	0.743	0.178
FocalStack+	0.829	0.764	0.678	0.807	0.142
Depth+	0.791	0.746	0.624	0.747	0.162
FocalStack *+	0.828	0.791	0.697	0.811	0.134
Depth *+	0.834	0.794	0.674	0.828	0.141
MAE Filter	**0.847**	**0.812**	**0.720**	**0.840**	**0.124**

* Optimized by CDCA model.

## Data Availability

Not applicable.
